# Effects of Mosquito Microbiota on the Survival Cost and Development Success of Avian *Plasmodium*

**DOI:** 10.3389/fmicb.2020.562220

**Published:** 2021-01-13

**Authors:** Josué Martínez-de la Puente, Rafael Gutiérrez-López, Alazne Díez-Fernández, Ramón C. Soriguer, Isabel Moreno-Indias, Jordi Figuerola

**Affiliations:** ^1^Estación Biológica de Doñana (EBD-CSIC), Seville, Spain; ^2^Centro de Investigación Biomédica en Red de Epidemiología y Salud Pública (CIBERESP), Madrid, Spain; ^3^Department of Endocrinology and Nutrition, Virgen de la Victoria University Hospital, Instituto de Investigación Biomédica de Málaga (IBIMA), University of Malaga, Málaga, Spain; ^4^Centro de Investigación Biomédica en Red de Fisiopatología de la Obesidad y la Nutrición (CIBEROBN), Madrid, Spain

**Keywords:** *Culex pipiens*, ecology-diseases, malaria, microbiome, parasite transmission, vector-borne pathogens, virulence

## Abstract

Both intrinsic and extrinsic factors affect the capacity of mosquitoes for the transmission of vector-borne pathogens. Among them, mosquito microbiota may play a key role determining the development of pathogens in mosquitoes and the cost of infections. Here, we used a wild avian malaria-mosquito assemblage model to experimentally test the role of vector microbiota on the cost of infection and their consequences for parasite development. To do so, a cohort of *Culex pipiens* mosquitoes were treated with antibiotics, including gentamicin sulfate and penicillin-streptomycin, to alter their microbiota, and other cohort was treated with sterilized water as controls. Subsequently, both cohorts were allowed to feed on *Plasmodium* infected or uninfected house sparrows (*Passer domesticus*). The antibiotic treatment significantly increased the survival rate of mosquitoes fed on infected birds while this was not the case of mosquitoes fed on uninfected birds. Additionally, a higher prevalence of *Plasmodium* in the saliva of mosquitoes was found in antibiotic treated mosquitoes than in mosquitoes of the control group at 20 days post exposure (dpe). Analyses of the microbiota of a subsample of mosquitoes at 20 dpe suggest that although the microbiota diversity did not differ between individuals of the two treatments, microbiota in control mosquitoes had a higher number of unique features and enriched in biochemical pathways related to the immune system than antibiotic treated ones. In sum, this study provides support for the role of mosquito microbiota on mosquito survival and the presence of parasite DNA in their saliva.

## Introduction

The vectorial capacity of mosquitoes, that describes the potential of a vector to transmit a pathogen, is driven by four major parameters including the blood feeding behavior of the insects, the ability of the pathogen to develop in the insects, the latency time, and the cost induced by pathogens in vector longevity (Macdonald, 1955; Rund et al., 2016). Both intrinsic and extrinsic factors, including behavioral, ecological and environmental variables, affect the capacity of mosquitoes for the transmission of mosquito-borne pathogens (Lefèvre et al., 2013). Among them, mosquito microbiota has been identified as a key component (Weiss and Aksoy, 2011) being involved in many biological processes of mosquitoes (Huang et al., 2020), finally determining the development of pathogens in vectors and the cost of infections (Guégan et al., 2018; Martínez-de la Puente et al., 2018). Mosquito microbiota affects the development of pathogens in the mosquitoes throughout different processes including the production of metabolites directly impairing parasite survival in the midgut and by stimulating the insect immunological responses (Dong et al., 2009; Romoli and Gendrin, 2018; Huang et al., 2020). Mosquito microbiota may reduce the success of parasite development, at least, in the *Anopheles*-human *Plasmodium* assemblages studied (Romoli and Gendrin, 2018), but contradictory results could be expected depending of pathogen-mosquito’s microbiota assemblages tested (Mideo, 2009; Romoli and Gendrin, 2018; Guégan et al., 2018). However, contrary to the case of parasites affecting humans, the role of mosquito microbiota on the transmission of vector-borne pathogens affecting wildlife has been traditionally neglected, as in the case of avian *Plasmodium* (Martínez-de la Puente et al., 2018).

Avian malaria parasites of the genus *Plasmodium* are mosquito-borne parasites naturally circulating between birds and mosquitoes. These parasites are considered excellent models for studies on the determinants of the ecology and evolution of the transmission dynamics of malarial parasites (Rivero and Gandon, 2018). The life cycle of avian *Plasmodium* parasites includes different phases in the vertebrate and invertebrate hosts. To be efficiently transmitted, a competent mosquito vector needs to feed on blood of an infected bird and, after parasite development in the mosquito, the insect may inoculate the parasite infective forms, the sporozoites, to a new host (Valkiūnas, 2005). Mosquitoes of different genera are competent vectors of avian malaria parasites, with those of the *Culex* genus playing a central role in their transmission (Santiago-Alarcón et al., 2012; Gutiérrez-López et al., 2020). Avian malaria infections in wild birds reduced survival and fitness of infected individuals (Merino et al., 2000; Asghar et al., 2015). In some cases, avian *Plasmodium* has dramatically affected bird populations. For instance, the widespread *Plasmodium relictum* is featured on the widely cited list of “100 of the World’s Worst Invaders” (Lowe et al., 2000) being considered a major cause of the decline of the populations of many avian species after its introduction in Hawaii (van Riper et al., 1986). Contrary to the case of their effects on vertebrate hosts, factors determining the interaction between mosquitoes and avian *Plasmodium* and their consequences for parasite amplification have been poorly investigated.

Here, we used a wild mosquito-*Plasmodium* assemblage to experimentally test the role of *Culex pipiens* microbiota on the mosquito survival and parasite development, two components determining the vectorial capacity of mosquitoes. Most studies conducted in this topic have used mosquito colonies to analyze the role of parasite microbiota. This may simplify the insect microbiota with respect to those present in wild mosquitoes, thus alternative models for the study of the mosquito and its microbiota are required (Romoli and Gendrin, 2018). We used *Cx. pipiens* mosquitoes raised in the laboratory from field collected larvae. This mosquito species was selected based on its ornithophilic behavior, frequently interacting with avian *Plasmodium* parasites infecting wild birds (Martínez-de la Puente et al., 2016, 2020; Rivero and Gandon, 2018). In addition, this mosquito is considered a competent vector for the transmission of different avian *Plasmodium* species (Santiago-Alarcón et al., 2012; Gutiérrez-López et al., 2020), thus playing a central role in the epidemiology of these pathogens under natural conditions.

## Materials and Methods

### Mosquito Sampling and Antibiotic Treatment

During 2018, mosquito larvae were collected in two close localities of the province of Huelva (Spain) according to the availability of mosquito breeding areas. Mosquito larvae were transferred to the laboratory where they were kept in plastic trays with water from the original breeding sites and fed with Mikrozell (20 ml/22 g; Hobby Mikrozell; Dohse Aquaristik GmbH & Co. KG, D-53501, Gelsdorf, Germany). Insects were maintained in a climatic chamber at constant conditions (temperature: 28°C, relative humidity (RH): 65–70%, light/dark cycle: 12:12 h). After emergence, adult mosquitoes were placed in insect cages (BugDorm-43030F, 32.5 × 32.5 × 32.5 cm) and fed *ad libitum* with sterilized 10% sugar solution. Two to five days later, female *Cx. pipiens* mosquitoes were identified by morphology (Schaffner et al., 2001) and mosquitoes of the same age and breeding area were assigned to each of the treatments: control and antibiotic-treated mosquitoes. Control mosquitoes were fed with sterilized 10% sugar solution, while experimental mosquitoes were fed with sterilized 10% sugar solution with antibiotics, which were 15 μg gentamicin sulfate (Sigma-Aldrich, Stockholm, Sweden) and 10 units/10 μg of penicillin-streptomycin (Invitrogen, Carlsbad, CA, United States) per ml of water solution (Dong et al., 2009). Mosquitoes were allowed to feed on the antibiotic treated or the control sugar solution during seven days prior to their exposure to vertebrate hosts. The sugar solution with or without antibiotics were replaced by sterilized water 24 h prior to each blood feed trial (see below) and access to water was removed 12 h before blood feed trials began.

### Bird Sampling and Experimental Assays

Eighteen juvenile house sparrows (*Passer domesticus*) were captured using mist nets in San Juan del Puerto (Huelva, Spain) and were individually ringed. We only included in this study those birds with single infections by *Plasmodium* parasites or uninfected birds. All birds with evidence of infection by *Haemoproteus*, *Leucocytozoon* parasites or mixed infections were removed from the experiment to avoid any confounding effect on mosquito survival (Valkiûnas et al., 2014). Each bird was immobilized and placed in an insect cage containing ≈50–100 mosquitoes. Each bird was exposed to control and antibiotic treated mosquitoes of the same age and geographical (breeding area) origin. Birds were exposed to mosquitoes of each treatment during 45 min directly in the field under dark conditions. The order of exposure of each bird to mosquitoes from each control or experimental treatments was randomly assigned. After the experiment, birds were blood sampled from the jugular vein using sterile syringes (never exceeding 1% of body mass) and released in the same area. Back to the laboratory, mosquitoes with a recent blood meal in their abdomen were separated in a new box and maintained in a climatic chamber with *ad libitum* access to sterilized 10% sugar solution during the following 20 days. Each box contained only engorged mosquitoes from the same treatment and fed on the same individual bird. The mortality rate of mosquitoes was daily monitored. A subsample of 65.90% (*n* = 315) fed mosquitoes that survived until the end of the experiment were used to molecularly identify the presence of avian *Plasmodium*. All mosquitoes from boxes containing less than 21 alive mosquitoes at the end of the experiment were analyzed. However, we only analyzed between 20 and 21 individuals in those cases where a higher number of mosquitoes survived until the end of the experiment. This subsample was selected based on the impossibility to handle additional mosquitoes in the same day and to reduce the cost of molecular analyses, while provide reliable estimates of parasite prevalence (Jovani and Tella, 2006). From these mosquitoes, we isolated the saliva following Gutiérrez-López et al. (2019), and the head-thorax of each mosquito (containing the salivary glands) was separated from the abdomen using sterile tips. Samples were kept in the freezer at −80°C.

### Molecular Analyses

The MAXWELL^®^ 16 LEV Blood DNA Kit was used to extract the genomic DNA from blood samples and from the head-thoraxes of mosquitoes. The Qiagen DNeasy^®^ Kit Tissue and Blood (Qiagen, Hilden, Germany) was used to extract the DNA from mosquito saliva. Detection and lineage identification of parasites were conducted following Hellgren et al. (2004). The presence of amplicons was verified in 1.8% agarose gels and positive samples were sequenced using the Macrogen Inc. facilities (Madrid, Spain). Sequences were edited using the software Sequencher^TM^ v 4.9 (Gene Codes Corp.^©^ 1991–2009, Ann Arbor, MI 48108, United States) and assigned to parasite lineages/morphospecies after comparison with those deposited in GenBank (National Center for Biotechnology Information) and Malavi databases (Bensch et al., 2009).

To detect bacterial species in mosquito midguts, we analyzed the microbiota profile from 16 blood-fed mosquitoes 20 days after blood-feeding (days post exposure, dpe), including eight antibiotic-treated mosquitoes and eight control mosquitoes. Mosquitoes of both treatments (controls and antibiotic treated mosquitoes) from the two larval collection localities and feed on four bird individuals were included in this study. None of these 16 mosquitoes were infected by avian *Plasmodium* (i.e., absence of parasite DNA in the head-thorax). Mosquito surface was sterilized in 70% ethanol, then rinsed in sterile PBS solution, and midguts were dissected with sterilized forceps and tips on clean smears and, subsequently stored individually in sterile water at −80°C. DNA extraction from each midgut was done using the QIAamp DNA stool Mini kit (Qiagen, Hilden, Germany) following the manufacturer’s instructions. DNA concentration and purity were estimated with a Nanodrop spectrophotometer (Nanodrop Technologies, Wilmington, DE, United States). Libraries from midguts were built with the Ion 16S Metagenomics kit (Thermofisher), consisting of primer pools to amplify multiple variable regions (V2, 3, 4, 6–7, 8 and 9) of the 16S rRNA. After generating amplicons, the Ion PlusTM Fragment Library Kit (Thermofisher) was used to ligate barcoded adapters and synthesize libraries. Barcoded libraries from all the samples were pooled and templated on the automated Ion Chef system (Thermofisher) followed by a 400 bp sequencing on the Ion S5 (Thermofisher). Three samples from the antibiotic-treated mosquitoes and one sample from the control group were discarded for posterior analyses due to the low number of sequences obtained (<15,000 reads).

### Statistical Analyses

Cox’s proportional hazards mixed−effect models by maximum likelihood were used to assess the effect of antibiotic treatment on mosquito survival until 20 dpe. The exposure order of birds (birds exposed first to antibiotic-treated mosquitoes and later to control mosquitoes, or vice versa) were included as a fixed factor to control for potential effects of mosquito bites on *Plasmodium* development. Independent models were used for those mosquitoes fed on *Plasmodium* infected and uninfected birds. We used this approach in order to statistically control for the bird identity in the analyses (i.e., birds were either infected or uninfected). In addition, this procedure allows us to control for additional factors linked to bird identity but not considered in the analyses, which could affect the results (e.g., the phase of infection, the parasite intensity in the bird or the immunological / nutritional status of birds). Differences in the presence/absence of *Plasmodium* in the head-thorax or saliva of mosquitoes were analyzed using Generalized Linear Mixed Models (GLMMs) with binomial error and logit link function including the antibiotic treatment as a fixed factor and bird identity and exposure order as random terms. Statistical analyses were performed in R software 3.2.5 (R Core Team, 2016) with the package *lme4* (Bates et al., 2015).

The bacteria sequences obtained from mosquito midguts were translated into amplicon sequence variants (ASVs) using DADA2 (Callahan et al., 2016) within the microbiome analysis package QIIME2 2019.1^1^. The same package was used for diversity analysis and subsequent taxonomic analysis through clustering with VSEARCH function (Rognes et al., 2016) and the reference base Greengenes version 13_8 at 97% of identity. Weighted Unifrac distance was used for diversity analysis (Lozupone et al., 2011). Differential abundance analysis was assessed with ANCOM within QIIME2 (Mandal et al., 2015) and core features were compared with a Venn diagram with Venny 2.1.0 (Oliveros, 2007). PICRUSt 1.1.1 was used to infer the functional profiles of the microbial communities (Langille et al., 2013). We inferred the biochemical pathways of the microbiota found in mosquitoes of each treatment through the Kyoto Encyclopedia of Genes and Genomes (KEGG). This procedure allows to search for the potential role of identified microbiota affecting the physiological pathways on the host (i.e., mosquitoes). The KEGG ortholog predictions were calculated, and subsequently translated into KEGG Pathways. The Statistical Analysis of Metagenomic Profiles (STAMP) tool was used for the analysis of the KEGG Pathways (Parks et al., 2014).

## Results

**FIGURE 1:**
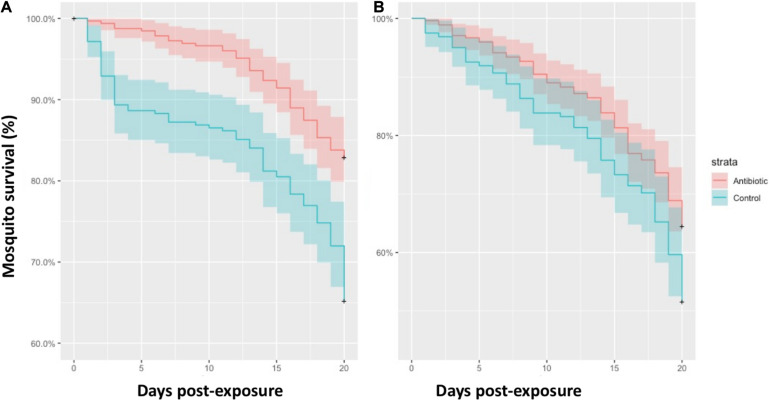
Survival rate of mosquitoes treated with antibiotics (red lines) and control mosquitoes (blue lines) fed on *Plasmodium* infected birds **(A)** and uninfected birds **(B)**. Colored areas represent standard errors.

Eight uninfected and ten *Plasmodium* infected birds corresponding to the *P. relictum* lineages SGS1 (*n* = 8) and GRW11 (*n* = 1) and the *Plasmodium* sp. lineage COLL1 (*n* = 1) were exposed to 2,250 mosquitoes. At the beginning of the experiment, 1,066 of these mosquitoes took a blood meal, including 632 mosquitoes fed on birds infected with *Plasmodium* and 434 mosquitoes fed on uninfected birds. For mosquitoes fed on *Plasmodium* infected birds, we found a higher survival to the 20 dpe of antibiotic-treated (271 out of 327; 82.87%) than control mosquitoes (207 out of 305; 67.87%) (Figure 1A; Cox model, treatment: *Z* = 4.76, *P* < 0.001; exposure order: *Z* = 0.68, *P* = 0.49). A similar trend was found for the case of mosquitoes fed on uninfected birds, although the effect of the antibiotic treatment on the survival of mosquitoes did not reach significance (Figure 1B; treatment: *Z* = 1.95, *P* = 0.052; exposure order: *Z* = −1.15, *P* = 0.25; antibiotic treated mosquitoes: 176/273, 64.47%; control mosquitoes: 83/161, 51.55%).

**FIGURE 2:**
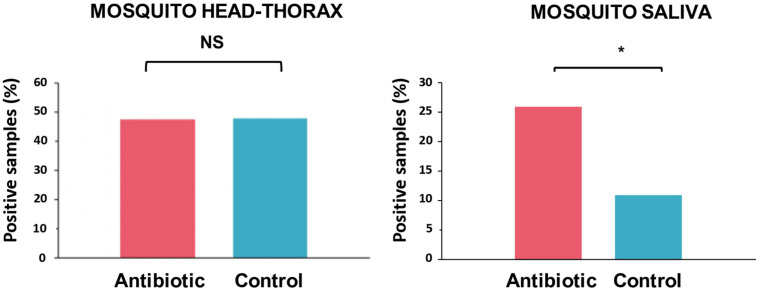
Percentage of the head-thoraxes and saliva samples with presence of *Plasmodium* DNA according to their experimental treatment: mosquitoes treated with antibiotics (red) and control mosquitoes (blue). Statistically significant differences are indicated with an asterisk (*). NS means non-significant differences.

The head-thorax of 315 out of 478 mosquitoes fed on infected birds that survived until 20 dpe were tested for the presence of avian *Plasmodium*. These analyses included 153 mosquitoes of the control group and 162 mosquitoes of the antibiotic-treated group. Of them, 150 (47.62%) were positive, including 73 mosquitoes treated as controls and 77 mosquitoes treated with antibiotics. The infection status of the head-thorax of mosquitoes fed on infected birds did not differ between treatments (Figure 2; *Z* = −0.69, *P* = 0.49). Of these mosquitoes with positive head-thoraxes, 20 individuals treated with antibiotics (*n* = 77; 25.97%) showed *Plasmodium* parasites in their saliva, while this was the case of only 8 (*n* = 73; 10.96%) mosquitoes treated as controls. Thus, a higher infection rate was found in the saliva of mosquitoes treated with antibiotics with respect to control ones (Figure 2; *Z* = −2.08, *P* = 0.037).

**FIGURE 3:**
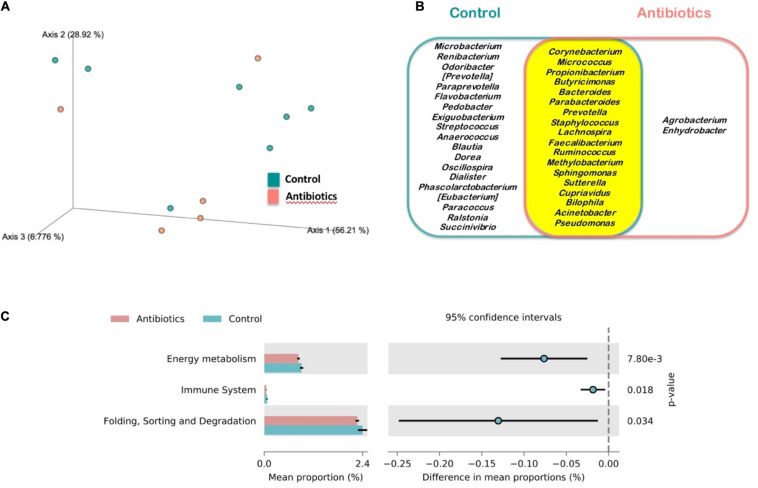
Microbiota analysis of mosquitoes treated with antibiotics (red) and control mosquitoes (blue). **(A)** Principal Coordinates Analysis (PCoA) of bacterial communities. **(B)** Venn diagram of the core microbiomes at genus level of the analyzed mosquitoes for the case of bacteria identified in control mosquitoes (blue), mosquitoes treated with antibiotics (red) and those shared by mosquitoes of both experimental groups (yellow). **(C)** Differentially abundant KEGG pathways identified at level 2 in both sampled groups.

Microbiota (core) features shared by at least 85% of the samples of a group were calculated to find the shared microbiome between experimental groups. A total of 18 out of 39 features, which were classified at genus level, were shared by both groups while 19 genera were exclusively found in control mosquitoes and only two in antibiotic-treated mosquitoes (Figure 3B). The complete list of bacteria found in mosquitoes is shown in Supplementary Table S1. However, no statistical differences were found neither in alpha (Shannon index; H = 0.798, *p* = 0.372) nor in beta diversity (weighted unifrac distance; pseudo-F = 0.764, *p* = 0.498) in mosquitoes of both treatments (Figure 3A). Moreover, the strict ANCOM analysis did not detect any compositional statistical difference between groups (data not shown). In spite of that, KEGG Pathways analysis revealed that microbiota from control mosquitoes was enriched in pathways related to energy metabolism, immune system and folding, sorting and degradation (Figure 3C).

## Discussion

Different studies have shown that microbiota affects the development of different pathogens on their vectors (Gendrin et al., 2015; Kalappa et al., 2018), although information on the impacts of mosquito microbiota on the development of protozoans of wild animals is scarce. To the best of our knowledge, we have tested for the first time the role of mosquito microbiota on the development and cost of infection (i.e., survival rate) of avian *Plasmodium* in its main vector *Cx. pipiens*. We found that antibiotic administration increased both the survival probability of mosquitoes and the presence of parasite DNA in their saliva after biting on naturally *Plasmodium*-infected birds.

*Plasmodium* infections may impact mosquito survival, although this effect may depend on the vector–parasite combinations studied or the methodological approaches used (e.g., the duration of the studies) (Ferguson and Read, 2002). In the case of mosquitoes infected by avian *Plasmodium* contradictory results have been reported (Martínez-de la Puente et al., 2018). For instance, while Gutiérrez-López et al. (2019) found experimental support for the negative effect of parasite infections on mosquito survival, other authors have found the opposite pattern (Vézilier et al., 2012) or, even, non-significant associations between mortality rate and parasite infection (Delhaye et al., 2016). Different intrinsic, including genetic differences between mosquitoes, and extrinsic factors may modulate the cost of avian malaria parasites in the vectors. These discrepancies could be partially explained by differences in the sugar concentration provided to the mosquitoes or the parasite species used or, even, to other factors including the parasite load of the bird donors (Martínez-de la Puente et al., 2018; Gutiérrez-López et al., 2020). These factors merit further research in order to identify their relevance on the effects of mosquito microbiota on parasite development and the cost of infections in mosquitoes. Our results indicate that mosquito microbiota also determine the cost imposed by the parasites on their vectors, a factor that has been traditionally neglected in studies on avian malaria parasites. Mosquito microbiota may have protective effects against parasite infections such as the inhibitory bioactivity of secreted enzymes or toxins and the mosquito physiological responses against parasites induced by their microbiota (Weiss and Aksoy, 2011; Smith et al., 2014). Thus, it could be expected a lower survival rate of mosquitoes fed on infected birds treated with antibiotics than controls. However, similarly to our case, Gendrin et al. (2015) found a positive effect of antibiotics on mosquito survival after feeding on blood infected with the rodent parasite *Plasmodium berghei*. Authors from this experimental study argued that the increase of the microbial populations following a mosquito blood meal might determine the observed pattern. This could also explain the results reported here and the lack of significant differences between the microbiota profiles of the respective groups. In addition, the immunological responses against the bacterial population grown could induce important costs for mosquitoes (Ahmed et al., 2002), finally affecting their longevity. Indeed, a higher immune system response, as well as a higher energy metabolism and activities related to the processing of genetic information, have been inferred within the control group from its microbiota KEGG pathways. Additionally, it is possible that the presence of particular bacteria in control mosquitoes increase their mortality rate, as in the case of mosquitoes exposed to *Chromobacterium* (Ramirez et al., 2014), although this genus was not found in the mosquitoes studied here. The antibiotic treatment did not significantly affect the survival probability of mosquitoes exposed to uninfected birds, although the same trend was found. Thus, the significant effects of the treatment on mosquito survival found in mosquitoes exposed to *Plasmodium* infected birds suggest a parasite-mediated effect of mosquito microbiota on survival. In this respect, in spite that we did not analyzed the presence of parasites in dead mosquitoes, it could be expected that infected mosquitoes were more likely to die during the course of the experiment (Valkiûnas et al., 2014).

We identified the presence of parasite DNA in the saliva of mosquitoes, and found that the antibiotic treatment affected the prevalence of avian *Plasmodium* DNA. In particular, we found a higher prevalence of parasites in mosquitoes supplemented with antibiotics than those treated as controls, suggesting that mosquito microbiota affected negatively the development of the parasites in their vectors. These results provide support to previous studies on other *Plasmodium*-vector assemblages using antibiotic alterations of mosquito microbiota (Romoli and Gendrin, 2018). For instance, the human malaria vector *Anopheles gambiae* fed on hosts treated with antibiotics were more susceptible to *Plasmodium* infections (Gendrin et al., 2015). More recently, Kalappa et al. (2018) found a higher prevalence of oocysts in *Anopheles stephensi* treated with antibiotics after their exposition to *P. berghei* infected mice. Our results support an effect of mosquito microbiota on parasite transmission in a novel study model. However, these differences were only evident when considering the presence of parasite DNA in the saliva of mosquitoes, but not in their head-thoraxes. Most studies on the vector capacity of mosquitoes for the transmission of avian malaria parasites are based on the amplification of parasite DNA in the head-thorax of individuals, whereas it is in the salivary glands of the mosquitoes where the infective forms of the parasites are accumulated. This method, although useful, may overestimate the capacity of vectors to transmit the parasites because DNA could be amplified from non-infective parasite forms present in the body even of non-competent insects (Valkiūnas, 2011).

Studies on the effects of mosquito microbiota on parasite transmission have largely used antibiotic treatments. These studies have found that mosquitoes supplemented with antibiotics reduce the bacterial load to undetectable levels (Dong et al., 2009), although antibiotics may fail to completely eliminate all the bacteria (Guégan et al., 2018). We failed to identify any significant effect of the antibiotic treatment in the diversity of the microbiota of mosquitoes at 20 dpe. However, mosquitoes treated with antibiotics had a lower number of unique features than those of the control group suggesting a simplification of their microbiota. In our study, mosquito larvae from both treatments were bred in the water from their breeding areas and were fed with the same diet, thus being colonized by similar bacteria (Linenberg et al., 2016; Guégan et al., 2018). In addition, the antibiotic treatment was provided to adult mosquitoes only prior to the bird exposure while mosquitoes of both treatments were supplemented with the same diet (i.e., sugar solution) after the blood meal. These could potentially affect the mosquito microbiota during this period and the absence of significant differences in the microbiota of mosquitoes of each treatment at the end of the experiment (20 dpe). It is also possible that the treatment had a homogeneous effect across taxa of the mosquito microbiota, explaining the absence of significant effects. However, our results suggest some simplification of the mosquito microbiota due to the antibiotic treatment as 19 bacteria genera appeared only in control mosquitoes while two were only found in antibiotic treated mosquitoes. Nevertheless, it is important to clarify that we are only looking at long-term effects of the treatment on the mosquito microbiota in a subset of only 12 mosquitoes. This is a limitation of our study that does not allow us to obtain further conclusions.

In summary, results from this study provide support for the importance of mosquito microbiota affecting two major parameters (survival rate and parasite development, measured as the presence of parasite DNA in the mosquito saliva) of models of vector transmission (i.e., Ross-MacDonald models). Differences in the microbiota exist between mosquito species and populations (Coon et al., 2016; Muturi et al., 2017; Duguma et al., 2019), which may affect their vector competence. These differences could explain, at least in part, the geographical differences found in the infection patterns between populations of wild birds, where a proportion of the variance is explained by the mosquito community present in the area and landscape configuration (Ferraguti et al., 2018). In addition, these results suggest that pollution of rivers by antibiotics used in human and animal health, which represent a worldwide problem especially in undeveloped countries (Danner et al., 2019), could also affect the epidemiology of mosquito-borne pathogens, such as avian *Plasmodium*. However, the antibiotic concentration used here is 500 times larger than those found in freshwaters, and consequently further experiments using antibiotic concentrations in the range found in antibiotic polluted areas is necessary to test this effect under more realistic conditions.

## Data Availability Statement

16S raw data was stored at the public repository SRA database (NCBI) with the BioProject PRJNA634467.

## Ethics Statement

The animal study was reviewed and approved by CSIC Ethics Committee.

## Author Contributions

JM-P, RS, and JF conceived and designed the study. JM-P, RG-L, and AD-F performed the experiments. JM-P, RG-L, and IM-I analyzed the samples and the data. JM-P led the writing of the manuscript. All authors contributed critically to the drafts and gave final approval for publication.

## Conflict of Interest

The authors declare that the research was conducted in the absence of any commercial or financial relationships that could be construed as a potential conflict of interest.

## Footnotes

1 www.qiime2.org
